# Lack of associations between betatrophin/ANGPTL8 level and C-peptide in type 2 diabetic subjects

**DOI:** 10.1186/s12933-015-0277-1

**Published:** 2015-08-20

**Authors:** Mohamed Abu-Farha, Jehad Abubaker, Fiona Noronha, Irina Al-Khairi, Preethi Cherian, Monira Alarouj, Abdullah Bennakhi, Naser Elkum

**Affiliations:** Biochemistry and Molecular Biology Unit, Dasman Diabetes Institute, P.O. Box 1180, Dasman, 15462 Kuwait City, Kuwait; Dasman Diabetes Institute, P.O. Box 1180, Dasman, 15462 Kuwait City, Kuwait; Clinical Epidemiology, Sidra Medical and Research Center, P.O. Box 26999, Doha, Qatar

## Abstract

**Background:**

Betatrophin has been suggested as an inducer of β-cell proliferation in mice in addition to its function in regulating triglyceride. Recent data showed that betatrophin was increased in Type 2 Diabetes (T2D), however, its ability to induce insulin production has been questioned. We hypothesized that the increased betatrophin in T2D is not affecting insulin production from β-cells. To test this hypothesis, we investigated the association between betatrophin and C-peptide level in humans, which acts as a measure of endogenous insulin production from β-cells.

**Methods:**

This study was designed to examine the association between plasma betatrophin level and C-peptide in 749 T2D and non-diabetics.

**Results:**

Betatrophin and C-peptide levels were higher in T2D subjects compared with non-diabetics subjects. Betatrophin showed strong correlation with C-peptide in non-diabetics subjects (*r* = 0.28, *p* = < 0.0001). No association between betatrophin and C-peptide were observed in T2D subjects (*r* = 0.07, *p* = 0.3366). Dividing obese and non-obese subjects into tertiles according to betatrophin level showed significantly higher C-peptide levels at higher tertiles of betatrophin in obese non-diabetics subjects *P*-trend = 0.0046. On the other hand, C-peptide level was significantly higher in subject with higher betatrophin level in non-diabetics subjects across all age groups but not in T2D subjects. Multiple logistic regression models adjusted for age, BMI, gender, ethnicity as well as C-peptide level showed that subjects in the highest tertiles of betatrophin had higher odds of having T2D [odd ratio (OR) = 7.3, 95 % confidence interval (CI) 4.0–13.3].

**Conclusion:**

Increased betatrophin level in obese subjects is correlated with an increase in C-peptide level; which is possibly caused by the increased insulin resistance. On the other hand, no correlation is observed between increased betatrophin level and C-peptide in T2D subjects. In conclusion, the increased betatrophin in T2D subject does not cause any increase in insulin production as indicated by C-peptide level.

## Background

Regeneration of insulin producing β-cells in diabetic patients has been cherished as the ultimate treatment for type 1 diabetes (T1D) and T2D [1–4]. It is well documented that β-cell replication slows down dramatically under physiological conditions in adult humans and rodents [5, 6]. Enhanced β-cell proliferation occurs naturally during pregnancy [5, 7] as well as obesity induced states of insulin resistance [8, 9].The recent discovery of betatrophin as a potential inducer of β-cell proliferation in mice in response to insulin resistance has revived the hope for achieving this goal [10, 11]. Betatrophin was initially identified as an ANGPTL protein family member and named ANGPTL8 due to its high similarity to angiopoetin like proteins [12, 13]. Many studies supported this role of betatrophin in lipid metabolism and adipocytes differentiation [12–18]. In an exciting twist, Yi et al. has shown that betatrophin, a liver and adipose tissue derived hormone, was able to induce β-cell proliferation in insulin resistant mouse model [11]. This finding caused huge excitement in the field of beta-cell regeneration as a potential alternative treatment for diabetes [10, 19]. Nonetheless, Jiao et al. demonstrated that betatrophin was able to cause strong induction of the β-cells proliferation in mice but not humans [20]. The authors questioned the ability of betatrophin to induce β-cell proliferation but highlighted a major caveat in their data which was the ability of the mouse betatrophin to act on the human receptor [20]. Recent reports have challenged the ability of betatrophin to induce β-cell proliferation [21, 22]. Gusarova et al. showed that β-cells lacking both copies of betatrophin were producing insulin normally under insulin resistance conditions [22].

A number of studies showed that betatrophin level was increased in type 1 [23], T2D [23–28] and gestational diabetes [29]. Using a large sample cohort we have recently showed that betatrophin level was increased in T2D, however, it was not affecting fasting blood glucose (FBG) or insulin production in T2D subjects [30]. As a result, a major question has been raised regarding the function of betatrophin in T2D and its ability to induce β-cell proliferation as well as its physiological role in humans [19, 20, 22, 31]. To answer this question, we designed a study to look at the association between betatrophin level and C-peptide in diabetic and non-diabetic subjects to understand the effect of betatrophin on the secretion of insulin. C-peptide is a cleavage product of proinsulin that is produced by the pancreatic β-cells at an equimolar amounts to insulin [32]. It is commonly used to assess β-cell function due to its longer half life and its ability to indicate endogenous insulin production in patients taking insulin treatments [32].

## Research design and methods

### Study participants and anthropometric and physical measurements

This is a study was performed on 749 adult (>18 years old) South Asians (Indians and Pakistanis) and Arabs living in Kuwait. The study was designed as cross-sectional. Samples have been randomly collected from multi-ethnic subjects living in Kuwait as described previously [33, 34]. Samples were continuously collected. Briefly, study participants suffering from any kind of infection as well as subjects younger than 18 and older than 65 were excluded. The non-diabetic subjects were then selected as subjects without disease and not taking any medications. Most subjects were taking oral hypoglycaemic agents alone (metformin, sulfonylurea and dipeptidyl peptidase-4 inhibitors), insulin alone or insulin with oral hypoglycaemic agents. A small number of subjects were using exercise and diet to control their diabetes. In addition, diabetic patients were taking lipid lowering, anti-Asthma and anti-hypertensive drugs. Subjects with cardiovascular diseases were excluded from the study. No treatment was received before sampling. Even though the selected participants had similar population characteristics as the total study population in terms of age, gender, FBG, body mass index (BMI), blood pressure (BP) and lipid profile, the T2D and the non-diabetic group were not exactly matched posing as one of the limitations of this study. The study conformed to the principles outlined in the Declaration of Helsinki and in accordance with the approved guidelines. The study was approved by the Ethical Review Committee at Dasman Diabetes Institute (DDI). An informed written consent was obtained from all the participants before their enrolment in the study.

Physical and anthropometric measurements included body weight, height, waist circumference (WC) were measured as described previously [33, 34]. Height and weight were measured, with participants wearing light indoor clothing and barefooted, using calibrated portable electronic weighing scales and portable inflexible height measuring bars. WC was measured using constant tension tape at the end of a normal exhalation, with arms relaxed at the sides, at the highest point of the iliac crest and at the mid-axillary line. BMI was calculated using the standard BMI formula: body weight (in kilograms) divided by height (in meters squared).

### Laboratory measurements

Blood samples were obtained after fasting overnight for at least 10 h and analyzed for FBG, HbA1c, fasting insulin, and lipid profiles that included triglyceride (TG), Total cholesterol (TC), low density lipoprotein (LDL) and high density lipoprotein (HDL). Glucose and lipid profiles were measured on the Siemens Dimension RXL chemistry analyzer (Diamond Diagnostics, Holliston, MA, USA). HbA1c was determined using the VariantTM device (BioRad, Hercules, CA, USA). All laboratory tests were performed by certified technicians at the clinical laboratories of DDI using the Ministry of Health approved methods and quality standards. Insulin resistance was calculated using the homeostatic model assessment-insulin resistance (HOMA-IR) formula: FBG (mmol/l) × fasting insulin (mU/l)/22.5.

### Diabetes diagnosis and guidelines

The current recommendations and updated guidelines for the definition, diagnosis and classification of T2D, published by the International Diabetes Federation (IDF), have been used as described previously [35]. Diabetes was defined by fasting plasma glucose ≥7 mmol/l, under treatment, or self-reporting of previously diagnosed T2D [35]. Impaired fasting glucose (IFG) was defined by fasting blood glucose values ≥5.6 and <7 mmol/l.

### ELISA betatrophin and C-peptide level

To measure metabolic markers, blood was drawn into EDTA tubes. Plasma was obtained after centrifugation, aliquoted and then stored at −80 °C. Betatrophin concentration was determined using ELISA (Wuhan EIAAB) as reported previously [24, 36, 37]. The assay showed linearity at dilutions ranging from 1:10 to 1:40. No significant cross reactivity with other proteins has been observed. Intra-assay coefficients of variation were 1.2–3.8 %, while the inter-assay coefficients of variation were 6.8–10.2 %. C-peptide was measured using Mercodia Ultrasensitive C-peptide ELISA according to the manufacturer’s instructions (Mercodia, Uppsala, Sweden). Inter- and intra-assay coefficient of variation was <5 %.

### Statistical analysis

Normality tests were run to assess data distribution. Comparisons between subjects with T2D and without T2D were made by Student’s *t* test or Wilcoxon test for non-parametric analyses in variables with non-normal distribution. To assess the difference in categorical variables between subjects with and without T2D, a Chi Squared test was used. Spearman’s correlation coefficients were estimated to determine associations between betatrophin and anthropometric measurements and biochemical variables. Subjects were classified into tertiles based on their circulating betatrophin levels in the overall population. Betatrophin tertile values for non-obese (BMI < 30) non-diabetics and T2D subjects are T1: ≤1162.5 pg/mL, T2: 1162.5 ≤ 1881.9 pg/mL, T3: > 1881.9 pg/mL). Betatrophin tertile values for the obese group are T1: ≤ 1273.4 pg/mL, T2: 1273.4 ≤ 1881.9 pg/mL, T3: > 1881.9 pg/mL. Betatrophin tertile values in subjects less than 40 years old stratified according to betatrophin level in non-diabetics and T2D subjects are T1: ≤ 567.6 pg/mL, T2: 567.6 ≤ 763.5 pg/mL, T3: > 763.5 pg/mL). B: Betatrophin tertiles in non-diabetics and T2D subjects between 40 and 50 years old are T1: ≤ 743.2 pg/mL, T2: 743.2 ≤ 1070.7 pg/mL, T3: > 1070.7 pg/mL). Betatrophin tertiles in non-diabetics and T2D subjects older than 50 years old are T1: ≤ 957.7 pg/mL, T2: 957.7 ≤ 1406.3 pg/mL, T3: > 1406.3 pg/mL).

A multivariable logistic regression analysis was performed to estimate odds ratios (ORs) adjusted for covariates and to assess the predictive effect of betatrophin on risk for T2D. All data are reported as Mean ± standard deviation (SD) and range, unless stated otherwise. Research Electronic Data Capture (REDCap) was used for data collections and data management. All statistical assessments were two-sided and considered to be significant when *P*-*value* < 0.05. All analyses were performed using SAS (version 9.2; SAS Institute, Cary, NC).

## Results

The clinical characteristics of the study population for both T2D and non-diabetics subjects are shown in Table 1. Our sample cohort was made of 749 subjects, 535 of which were non-diabetics and 214 were T2D. The average age of participants was 41.3 ± 10.1 years for non-diabetics subjects and 50.5 ± 9.8 years for T2D subjects. T2D subjects had higher BMI, waist/hip ratio, systolic BP, diastolic BP, FBG, HBA1C, insulin, HOMA-IR, TG, HDL (p < 0.05). TC did not show any significant changes between the two groups while LDL level was lower in T2D subjects. Betatrophin was higher in subjects with T2D relative to non-diabetics subjects [1710.1 (197.4–10972.1] pg/mL vs. 720.3 (59.5–9345.1) pg/mL) respectively. C-peptide was also higher in T2D subjects compared to non-diabetics subjects (744.1 ± 419.0 vs. 606.2 ± 300.1 pmol/L).

**Table 1:** Clinical and biochemical profiles for non-diabetics and T2D subjects

Variables	Normal (n = 535)	T2D (n = 214)	*P* value
Age (years)	41.3 ± 10.1	50.5 ± 9.8	<0.0001
Ethnicity			
Arab	285 (53.3 %)	110 (51.4 %)	
South Asian	250 (46.7 %)	104 (49.6 %)	
BMI (kg/m^2^)	29.36 ± 6.23	30.72 ± 5.59	0.0039
Waist/hip ratio	0.91 ± 0.11	0.95 ± 0.06	<0.0001
Systolic (mmHg)	128.6 ± 17.9	138.7 ± 19.7	<0.0001
Diastolic (mmHg)	78.9 ± 12.2	81.4 ± 11.4	0.0083
FBG (mmol/L)	5.02 ± 0.58	8.91 ± 3.60	<0.0001
HBA1C (DCCT %)	5.47 ± 0.70	7.79 ± 2.09	<0.0001
Insulin (mU/L)	9.72 ± 6.23	20.43 ± 41.87	0.0003
HOMAIR	2.22 ± 1.60	8.03 ± 17.66	<0.0001
Total cholesterol (mmol/L)	5.17 ± 1.09	5.13 ± 1.07	0.5694
Triglycerides (mmol/L)	1.50 ± 0.87	2.04 ± 1.47	<0.0001
HDL cholesterol (mmol/L)	1.14 ± 0.33	1.06 ± 0.28	0.0022
LDL cholesterol (mmol/L)	3.40 ± 0.99	3.21 ± 0.94	0.0154
Betatrophin (pg/mL)	720.3 (59.5–9345.1)	(1710.1 (197.4–10972.1)	<0.0001
C peptide	606.2 ± 300.1	744.1 ± 419.0	<0.0001

Results are reported as Mean ± SD except for non-normally distributed betatrophin that are presented as Median (range). Diabetes: fasting plasma glucose ≥ 7 mmol/l, under treatment, or self-reported of previously diagnosed T2D

Partial Spearman correlation coefficients were adjusted for age, gender, and ethnicity. Spearman’s correlation showed significant positive association in non-diabetic subjects between betatrophin and age (*r* = 0.50, *p* = < 0.0001), BMI (*r* = 0.13, *p* = 0.0018), FBG (*r* = 0.19, *p* = < 0.0001), and insulin (*r* = 0.11, *p* = 0.00121) as shown in Table 2. Strong positive correlation was also observed between betatrophin and C-peptide in non-diabetics subjects (*r* = 0.28, *p* = < 0.0001). On the other hand, subjects with T2D also showed positive correlation with age (*r* = 0.48, *p* = < 0.0001). No association was observed between betatrophin and FBG, insulin or C-peptide in T2D subjects (Table 2). On the other hand, in non-diabetics subjects, C-peptide showed strong positive association with age (*r* = 0.17, *p* = < 0.0001), BMI (*r* = 0.44, *p* = < 0.0001), FBG (*r* = 0.35, *p* = < 0.0001) and insulin (*r* = 0.72, *p* = < 0.0001). In T2D subjects, C-peptide showed positive association with BMI (*r* = 0.31, *p* = < 0.0001) and insulin (*r* = 0.35, *p* = < 0.0001) (Table 2).

**Table 2:** Partial Spearman correlations between betatrophin levels and diabetes/metabolic risk factors

Variable	All		T2D		Non-diabetics	
	Betatrophin	C-peptide	Betatrophin	C-peptide	Betatrophin	C-peptide
Age	0.56 (<0.0001)	0.17 (<0.0001)	0.48 (<0.0001)	−0.02 (0.8049)	0.50 (<0.0001)	0.17 (<0.0001)
BMI	0.17 (<0.0001)	0.41 (<0.0001)	0.13 (0.0535)	0.31 (<0.0001)	0.13 (00.0018)	0.44 (<0.0001)
FBG	0.40 (<0.0001)	0.35 (<0.0001)	−0.02 (0.8231)	0.09 (0.1965)	0.19 (<0.0001)	0.35 (<0.0001)
Insulin	0.17 (<0.0001)	0.63 (<0.0001)	−0.03 (0.7059)	0.39 (<0.0001)	0.11 (0.0121)	0.72 (<0.0001)
Cpeptide	0.26 (<0.0001)		0.07 (0.3366)		0.28 (<0.0001)	
Betatrophin		0.27 (<0.0001)		0.07 (0.3366)		0.28 (<0.0001)

Diabetes: fasting plasma glucose ≥7 mmol/L, under treatment, or self-reported of previously diagnosed T2D; waist/hip ratio. Duration of diabetes was calculated for subjects with diabetes. Partial Spearman correlation coefficients were adjusted for age, gender, and ethnicity

*BMI* body mass index, *FBG* fasting blood glucose

**Fig. 1:**
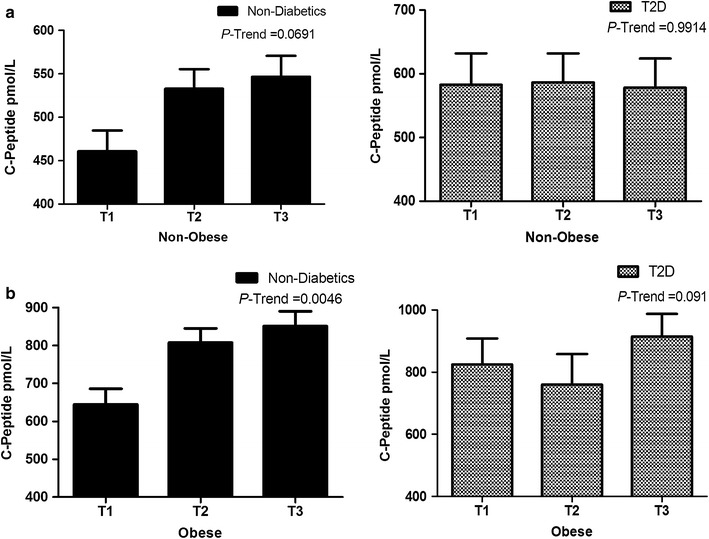
C-peptide level according to betatrophin tertiles in non-diabetics and T2D subjects. **a** Level of C-peptide in non-obese (BMI < 30) non-diabetics and T2D subjects at different betatrophin tertiles (betatrophin tertile values for the non-obese group are T1: ≤1162.5 pg/mL, T2: 1162.5 ≤ 1881.9 pg/mL, T3: > 1881.9 pg/mL). **b** C-peptide level in obese (BMI ≥ 30) non-diabetics and T2D subjects at different betatrophin tertiles (betatrophin tertile values for the obese group are T1: ≤1273.4 pg/mL, T2: 1273.4 ≤ 1881.9 pg/mL, T3: > 1881.9 pg/mL)

In order to study the effect of betatrophin on C-peptide level, our population was divided into tertiles according to betatrophin level. Betatrophin was reported as least square means adjusted for age, gender and ethnicity as shown in Fig. 1. Figure 1 shows a significant increase in C-peptide level at higher tertiles of betatrophin in non-diabetics obese subjects *P*-trend = 0.0046. A similar trend was also observed in the T2D obese subjects albeit not statistically significant *P*-trend = 0.0983. Non-obese, non-diabetics showed a slight increase in C-peptide level in higher tertiles of betatrophin *P*-trend = 0.0691. However, the non-obese T2D subject did not show any increase in C-peptide level in concordance with the increase in betatrophin level as shown in Fig. 1 (*P*-trend = 0.9914).

**Fig. 2:**
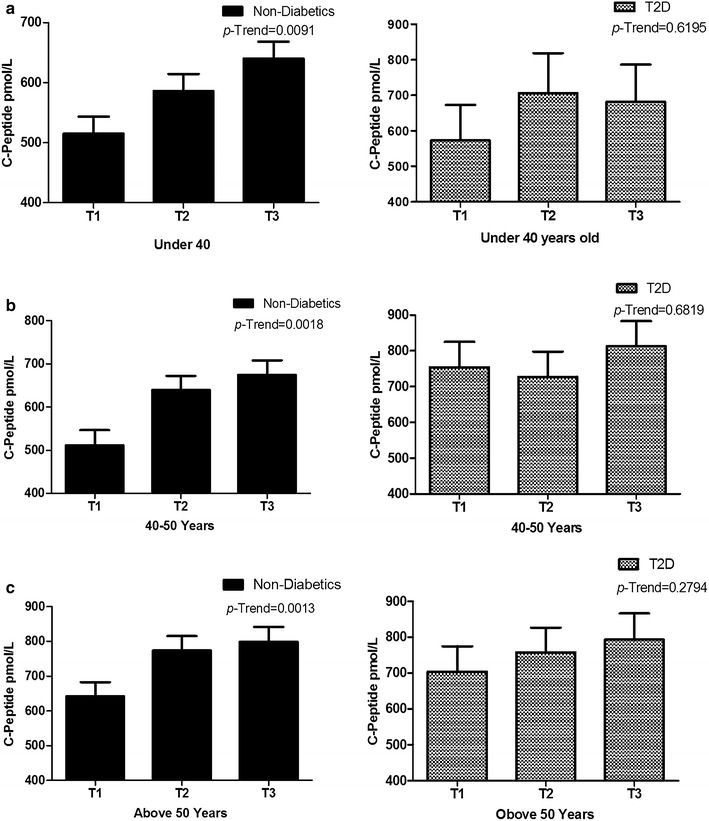
C-peptide level for different age groups at different betatrophin tertiles. **a** C-peptide level in subjects less than 40 years old stratified according to betatrophin level in non-diabetics and T2D subjects (betatrophin tertile values are T1: ≤567.6 pg/mL, T2: 567.6 ≤ 763.5 pg/mL, T3: >763.5 pg/mL). **b** C-peptide level in subjects that are between 40 and 50 years old according to betatrophin tertiles in non-diabetics and T2D subjects (betatrophin tertile values are T1: ≤743.2 pg/mL, T2: 743.2 ≤ 1070.7 pg/mL, T3: >1070.7 pg/mL). **c** C-peptide level in subject that are older than 50 years old according to betatrophin tertiles in non-diabetics and T2D subjects (betatrophin tertile values are T1: ≤957.7 pg/mL, T2: 957.7 ≤ 1406.3 pg/mL, T3: >1406.3 pg/mL)

Since betatrophin is strongly correlated with age, diabetic and non-diabetic subjects were divided into three age groups as follows (<40 years, 40–50 years and >50 years old). Level of C-peptide in age-, gender- and ethnicity-adjusted least square means tertiles of betatrophin are given in Fig. 2 for the different age groups. Figure 2 shows that C-peptide level in age group <40 years is increased in concordance with increased betatrophin in the non-diabetics group but not the T2D subjects *P*-trend = 0.0091 and *P*-trend = 0.6195 respectively. A similar trend was also observed for the other two age groups (40–50 years and >50 years old) where C-peptide was increasing at higher levels of betatrophin in non-diabetics subjects only. Taken together, our data show that the increased betatrophin level in the diabetics is not affecting the C-peptide level across all age groups.

Multiple logistic regression analysis of betatrophin and C-peptide showed that in the unadjusted model, subjects in the highest tertile of betatrophin were more likely to have T2D (OR = 16.2, 95 % CI 9.5–27.5) (Table 3). After adjustment for age, BMI, gender and ethnicity in Model 2, subjects in the highest tertile of betatrophin had higher odds of having T2D (OR = 7.6, 95 % CI 4.2–13.3). Further adjustment for C-peptide in Model 3 moderately attenuated the association, none the less, subjects in the highest tertile still had higher odds of having T2D (OR = 7.3, 95 % CI 4.0–13.3) (*P*-trend < 0.0001). Multiple logistic regression analysis showed that, in the unadjusted model 1, subjects in the highest tertile of C-peptide had higher odds of having T2D (OR = 2.4, 95 % CI 1.6-3–3.5). After adjusting for age, BMI, gender and ethnicity in Model 2 or model 2 + betatrophin in model 3, no significant OR were achieved (Table 3).

**Table 3:** OR (95 % CI) by multiple logistic regression models for diabetes in relation to betatrophin and C-peptide

Models	Betatrophin (n = 749)			*P*-trend	C-peptide (n = 749)			*P*-trend
	T1, n = 247, T2D = 19	T2, n = 253, T2D = 52	T3, n = 249, T2D = 143		T1, n = 249, T2D = 51	T2, n = 251, T2D = 69	T3, n = 249, T2D = 94	
Model 1	1	3.1 (1.8–5.4)	16.2 (9.5–27.5)	<0.0001	1	1.5 (1.0–2.2)	2.4 (1.6–3.5)	0.0001
Model 2	1	1.9 (1.1–3.4)	7.6 (4.2–13.8)	<0.0001	1	1.2 (0.7–1.9)	1.6 (0.9–2.5)	0.1778
Model 3	1	1.8 (1.0–3.3)	7.3 (4.0–13.3)	<0.0001	1	1.1 (0.7–1.8)	1.3 (0.8–2.1)	0.6698

Model 1 unadjusted; Model 2 adjusted for age, BMI, gender and ethnicity; Model 3 adjusted for betatrophin + C-peptide + Model 2

Tertile values of C-peptide are expressed as T1 (<468.3), T2 (468.3–707.5), and T3 (>707.5). Tertile values of betatrophin are T1 (<743.24), T2 (743.2–1229.2), and T3 (>1229.2)

## Discussion

This study investigated the association between the increased betatrophin level in T2D subjects and insulin production as indicated by the C-peptide level. Even though, we show strong correlation between the level of betatrophin and C-peptide in the non-diabetics group, the T2D subjects did not show any correlation with C-peptide. On the other hand, obese subjects showed significant increase in C-peptide level at higher levels of betatrophin in non-diabetics subjects and a slight increase, yet not significant in the T2D subjects. Similarly the non-obese, non-diabetics had a slight increase in C-peptide level at higher betatrophin level unlike the non-obese T2D which did not show any significant increase in C-peptide level at higher levels of betatrophin. Increased betatrophin level across different age groups caused a significant increase in C-peptide level in non-diabetics subjects but not T2D subjects. Higher betatrophin levels were associated with 7.3 fold increase the odds of having T2D after adjusting for age, BMI, gender ethnicity and C-peptide. Taken together, our data clearly indicates that increased betatrophin level is associated with higher level of c-peptide in non-diabetics obese and older subjects but not T2D subjects.

### Effect of obesity on the association between betatrophin and C-peptide in subjects with or without diabetes

The recent identification of betatrophin role in β-cell proliferation in mice has caused an excitement as well as confusion in the community about its potential use in humans to treat T2D [10, 11, 19, 22, 38–40]. Our data as well as others showed that betatrophin is increased in T2D subjects [16, 30] as well as T1D subjects [36]. Animal studies have questioned the ability of betatrophin to induce β-cell proliferation in mice [22, 38]. To assess whether betatrophin had an impact on β-cells in humans, we used C-peptide as an indicator for endogenous insulin production. C-peptide is a cleavage protein released during insulin production from its precursor proinsulin [32, 41–45]. C-peptide is used clinically to assess β-cell function since it reflects the insulin production capabilities of the β-cells [32]. It also has a higher half life and very minimal liver clearance compared to insulin and reflects the endogenous insulin level in patients treated with insulin [32, 43, 45]. Our data clearly showed strong correlation between betatrophin and C-peptide in non-diabetics subjects. Obese subjects had a higher level of betatrophin and showed more increase in C-peptide level at increasing levels of betatrophin. This increase can be due to the state of insulin resistance in obese subjects caused by the chronic-low grade inflammation [46–48]. Even though β-cells replicate at a very low rate under normal condition, a state of insulin resistance can cause an increase in β-cell replication [4–6, 8]. Yi et al. ruled out the possibility that betatrophin was inducing insulin resistance to increase β-cell proliferation [11]. Using insulin tolerance test they did not observe any difference between betatrophin and control injected mice unlike the state of insulin resistance they observed in insulin receptor antagonist S961 treated mice [11]. As a result, it is possible that this increased state of insulin resistance is driving the increased betatrophin expression which in turn increases the production of insulin.

### Effect of age on the association between betatrophin and C-peptide in subjects with or without diabetes

Our work and that of others has shown that betatrophin level increases in older subjects [36]. It has been speculated that the increase in betatrophin level in older subjects is a mechanism for increasing insulin production since aging is associated with decreased insulin action. Age related factors have been suggested as the cause for the age related increased insulin resistance [49, 50]. These factors include age related changes such as decreased lean muscle mass, mitochondrial dysfunction, hormonal changes as well as increased oxidative stress and inflammation amongst many other changes [49]. The increased betatrophin level in older subjects can be a cellular mechanism to compensate for the increased insulin resistance in the non-diabetic subjects. Nonetheless, the large increase in betatrophin in T2D subjects was not able to affect C-peptide level and insulin secretion. In T2D subjects β-cells produce less insulin in response to stimuli compared with a normal cell, insulin response in stimulated β-cells from T2D subjects can be as low as 15 % of the normal [8].

It is possible that betatrophin acts as part of a mechanism to compensate for the increased insulin demand in insulin resistant subjects as we can see by the increased correlation with C-peptide in obese subjects compared to non-obese. However, betatrophin alone is not capable of compensating for this increase in insulin demand due to the development of a state of betatrophin resistance or other unknown factors that deem the β-cells unresponsive to the increased betatrophin level [30]. This was clearly demonstrated in our recent data that showed clear lack of association between betatrophin and FBG in T2D subjects [30]. Its effectiveness as an inducer of beta-cells has been also questioned as mentioned earlier by a number of reports that emphasises more its role in lipid metabolism [18]. On the other hand, this inability of the increased betatrophin in T2D subjects to compensate for the increased insulin resistance in older T2D subjects further supports the notion that beta-cell dysfunction is the main cause of T2D pathophysiology and not the age related factors mentioned earlier [49]. Overall, these findings uncover an important aspect about betatrophin and its future use as a β-cell stimulator to treat diabetes.

## Conclusion

In conclusion, our data shows strong correlation between betatrophin and C-peptide in non-diabetics but not T2D subjects suggesting that the increased production of betatrophin in T2D is not causing any increase in insulin production. In non-diabetics, increased betatrophin level is associated with increased C-peptide level highlighting the possibility that this increase in betatrophin is driven by insulin resistance to compensate for the increased insulin demand in obese subjects. Taken together, our data show that the increased betatrophin level in T2D subjects is not correlating with insulin production and its use as a diabetes treatment is questionable. However, it remains to be discovered whether betatrophin can be beneficial in modulating insulin production in insulin resistant non-diabetics obese subjects.
